# Hormonal balance, anovulatory cycles and luteal phase deficiency: exploring relationships between hematological variables, sex hormones and V̇O_2_max in athletes

**DOI:** 10.1530/RAF-24-0119

**Published:** 2025-04-28

**Authors:** Paula Recacha-Ponce, Pilar Suárez-Alcázar, Carlos Hernando, Pablo Salas-Medina, Maria Muriach, Pablo Baliño, Isabel Guisado-Cuadrado, Eladio Collado-Boira

**Affiliations:** ^1^ Nursing Department, University of Jaime I, Castellón de la Plana, Castellón, Spain; ^2^ Department of Education and Specific Didactics, University of Jaume I, Castellón de la Plana, Castellón, Spain; ^3^ Medicine Department, University of Jaime I, Castellón de la Plana, Castellón, Spain; ^4^ LFE Research Group, Department of Health and Human Performance. Faculty of Physical Activity and Sport Science-INEF. Universidad Politécnica de Madrid, Madrid, Spain

**Keywords:** menstrual cycle, anovulatory cycle, luteal phase deficiency, VO_2_max, iron, hemoglobin

## Abstract

**Graphical abstract:**

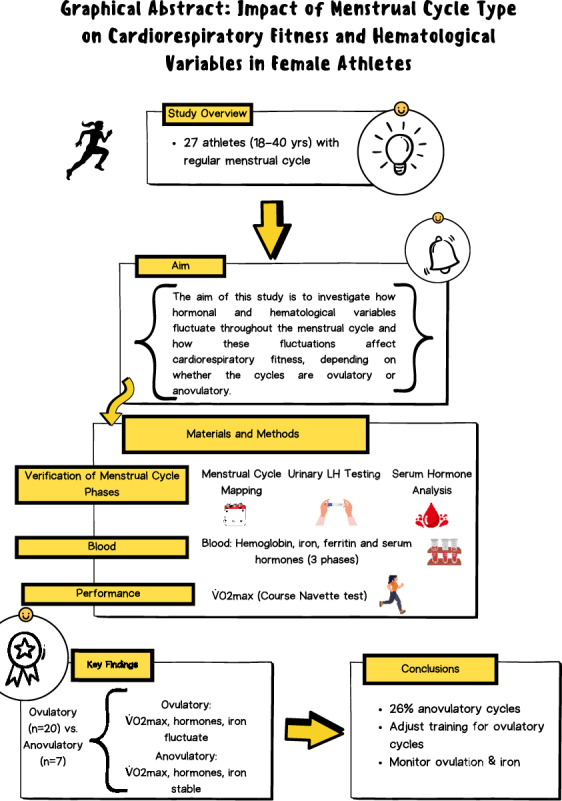

**Abstract:**

The impact of the menstrual cycle on physical fitness in athletes remains controversial in the scientific literature. Notable fluctuations in sex hormones occur at three key phases of the menstrual cycle, during which estrogen and progesterone levels vary significantly. In addition, the presence of regular bleeding does not ensure ovulation; therefore many women may not be aware that they have anovulatory cycles. These sex hormones can influence the physiology of women and can affect their level of cardiorespiratory performance depending on the phase of the menstrual cycle they are in. Method: Twenty-seven women aged 18–40 years with regular cycles were recruited. All participants had to be athletes classified as level II–III of the McKay *et al.* 2022 proposal based on training volume/physical activity metrics, among other variables. Cardiorespiratory fitness was indirectly assessed using V̇O_2_max measurements. Blood samples were collected on three occasions to determine the phase of the menstrual cycle by analyzing sex hormone levels. In addition, urine analyses were performed to detect ovulation, which was positive in all participants. To classify a cycle as ovulatory, progesterone levels must reach 16 nmol/L during the mid-luteal phase. However, it was observed that 26% of the sample did not reach this threshold, exhibiting anovulatory cycles or cycles with deficient luteal phases. Thus, two study groups were created: the ovulatory menstrual cycle group (*n* = 20) and the menstrual cycle group with deficient/anovulatory luteal phases (*n* = 7). These groups did not show statistically significant differences in age, weight, body mass index or V̇O_2_max during the bleeding phase (phase I). Female sex hormones did not show significant differences in the anovulatory cycle group, whereas they did show significant differences in the ovulatory cycle group. A high prevalence of female athletes with anovulatory menstrual cycles was observed. Women with ovulatory cycles experienced changes in their V̇O_2_max (*P* = 3.78E^−4^), in contrast to women with anovulatory cycles, who exhibited stable V̇O_2_max levels throughout their cycle (*P* = 0.638). Women with anovulatory menstrual cycles exhibit linear patterns of sex hormones throughout the menstrual cycle, which could lead to the maintenance of physical fitness throughout the cycle. In ovulatory cycles, it would be possible to polarize the training load according to the phase of the menstrual cycle. Monitoring ovulation, in addition to menstrual bleeding, is necessary to enhance knowledge about women’s reproductive health.

**Lay summary:**

The menstrual cycle may affect physical fitness in female athletes, but the evidence remains unclear. Hormones such as estrogen and progesterone fluctuate during different phases of the menstrual cycle and can influence performance. However, menstrual bleeding does not always indicate ovulation, and many women may not realize they are not ovulating or producing eggs. In our study, we analyzed 27 female athletes aged 18–40 with regular cycles, measuring cardiorespiratory fitness V̇O_2_max and hormone levels across the menstrual cycle. We found that 26% of participants were either not producing eggs or their womb lining was not thick enough to support a healthy cycle. These women showed consistent V̇O_2_max levels throughout the cycle, while those with ovulatory cycles** – where an egg is released – **exhibited variations in performance. These findings highlight the importance of monitoring ovulation, not just bleeding, to understand how the menstrual cycle impacts fitness and health. Tailoring training to menstrual phases could optimize performance for women with ovulatory cycles.

## Introduction

Female sex hormones play a central role in regulating fertility and reproduction, with secretion levels varying throughout the ovarian cycle, resulting in a range of systemic physiological effects (Oosthuyse & Bosch 2010). Due to these fluctuating hormone levels, much of the existing research tends to exclude women from studies (Cowley *et al.* 2021). This exclusion limits our understanding of how female sex hormones impact exercise responses and training adaptations in women (Elliott-Sale *et al.* 2021). In addition, even when studies do include women, poor methodological design can undermine findings. Proper monitoring of ovulation (through the determination of urinary LH) along with the levels of sex hormones (estrogens, progesterone, LH and follicle-stimulating hormone (FSH)) is essential to distinguish between different reproductive profiles accurately. Importantly, women with regular menstrual bleeding may not necessarily be ovulating (Liu *et al.* 2013). In exercise science, women who menstruate monthly are often labeled as ‘eumenorrheic’, which assumes uniform ovulatory and hormonal characteristics across all regular menstrual cycles. However, even menstrual cycles of typical length can vary significantly in their ovulatory features (Hackney *et al.* 2020), as regular bleeding does not guarantee ovulation (Liu *et al.* 2013). Without endocrine monitoring, there is a risk of generalizing findings across women with different reproductive profiles, including those with anovulatory cycles or luteal phase deficiencies. These non-ovulatory cycles often lack noticeable symptoms and are common among physically active women with high caloric demands due to training load (De Souza *et al.* 1998, Chen & Brzyski 1999, De Souza 2003, Sherman & Thompson 2004). Consequently, generalizing results without appropriate monitoring can be misleading.

Sex hormones, such as estrogen and progesterone, can influence athletic performance due to their impact on various bodily functions, including neuromuscular, sensorimotor, psychomotor, cognitive and psychological functions. These hormones affect multiple systems, including cardiovascular, respiratory, metabolic, body composition, thermoregulation and psychological factors. However, their effects are complex, as they can be antagonistic, synergistic or additive, with concentrations increasing with exercise. Furthermore, both endogenous and exogenous/synthetic hormones can alter physiological parameters, making their effects on athletic performance varied and difficult to determine (Jurkowski *et al.* 1978, Montagnani *et al.* 1992, Lebrun *et al.* 2020).

In this context, oxygen consumption (V̇O_2_) is a key physiological parameter that reflects the body’s ability to use oxygen per unit of time, directly linked to energy metabolism. The maximum oxygen consumption (V̇O_2_max), which indicates the body’s capacity to absorb, transport and utilize oxygen, depends on various factors such as cardiac output, hemoglobin levels and the concentration of red blood cells, among others. Hemoglobin plays a crucial role in oxygen transport, with iron being essential for its formation. Iron deficiencies, resulting from prolonged periods of insufficient iron, can lead to conditions such as iron-deficiency anemia, which negatively impacts physical endurance. Women, in particular, are at risk due to menstrual bleeding, which can lower iron levels, thus affecting performance (Zakin *et al.* 2002, Ainsworth *et al.* 2011, Caspersen *et al.* 1985).

V̇O_2_max is also influenced by factors such as training level and body composition. Athletes, especially those with better training, exhibit higher V̇O_2_max values. Hemoglobin and hematocrit levels are important factors in determining V̇O_2_max and, by extension, athletic performance, as they are responsible for oxygen transport in the blood. Variations in these factors during the menstrual cycle, due to fluctuations in iron and hemoglobin levels, can affect oxygen consumption and overall physical performance, highlighting the importance of managing these variables for optimal athletic outcomes (Friedmann *et al.* 2001, Prampero 2003, Besson *et al.* 2022).

However, differences may exist between ovulatory women and those with luteal phase deficiency or anovulatory cycles in how these variables behave. The aim of this study is to investigate how hormonal and hematological variables fluctuate throughout the menstrual cycle and how these fluctuations affect cardiorespiratory fitness, depending on whether the cycles are ovulatory or anovulatory.

## Materials and methods

### Ethical considerations

The study was conducted in accordance with the Declaration of Helsinki and was approved by the local ethics committee of the University Jaume I (CD/77/2020). All participants provided written consent before participating in the study. The study was registered at ClinicalTrials.gov (ID: NCT05576740).

### Participants

Women with natural menstrual cycles were recruited. All were classified within levels II and III of McKay *et al.*’s classification framework (McKay *et al.* 2022) based on training volume and performance metrics. Participants were recruited through social media. Interested women were informed and completed a questionnaire via the Qualtrics® platform. Selection was based on established inclusion and exclusion criteria.

Inclusion criteria were: i) healthy women, ii) aged between 18 and 40 years, iii) body mass index (BMI) ≥ 18.5, iv) classified within level II and III of McKay *et al.*’s framework, v) no alcohol or tobacco consumption, vi) women with regular menstrual cycles of 25–35 days over the past 6 months, and vii) no hormonal contraceptive use for at least 6 months prior.

**Figure 1:**
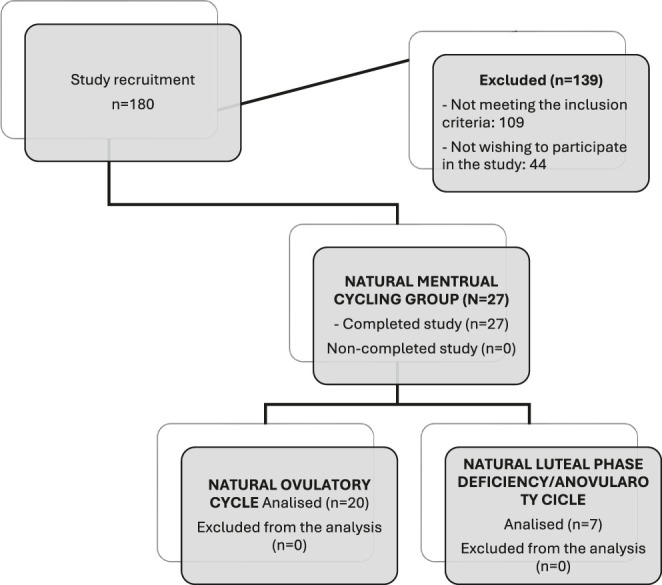
Flowchart to recruitment.

The total sample recruited exhibited physiological characteristics in their menstrual cycles. All selected women reported to the research team having had regular menstrual cycles for a minimum of 3 consecutive months, with cycle durations ranging from 21 to 35 days, and had not used hormonal contraceptives for at least 6 months before entering the study (Fig. 1).

This study employed a prospective cross-sectional cohort design. Two study groups were formed: the group of women with natural ovulatory menstrual cycles (OMCs) and the group with natural menstrual cycles having deficient/anovulatory luteal phases (AMC). Women with amenorrhea and those using hormonal contraception were excluded *a priori*.

### Experimental procedures

The order of tests was as follows for all participants.

#### Interview

Following the established testing protocol, during the first visit, acute symptomatology/illness, medication intake and the time of the last meal were initially verified. The timing of tests and prior exercise were adapted to participants’ work and sports schedules, ensuring that evaluations occurred at the same time of day and under the same preconditions.

#### Blood analysis

A venous blood sample was taken from the antecubital vein, with the woman lying down. For the hormone and iron samples, tubes without anticoagulant were used, while for the hemogram samples, EDTA tubes were used. The blood samples were allowed to rest for 10 min and then centrifuged. After centrifugation, the sample was transported to the laboratory for immediate analysis, always maintaining it in transport with ice. Blood samples were always collected before the physical test. Serum samples were processed using the Architect c-8000 system (Abbott Laboratories, USA) using chemiluminescence to determine LH, FSH, 17B-estradiol, progesterone, SHBG, testosterone and ferritin. For iron determination, the Konelab 30i equipment (USA) was used with a colorimetric and turbidimetric technique, respectively. Hemograms were processed from whole blood anticoagulated with tripotassium EDTA in a Horiba ABX Pentra XL 80 autoanalyzer (HORIBA Advanced Techno, Co., Ltd, Japan). Hemograms were processed after extraction, and serums were refrigerated until processed (never more than 24 h) (Recacha-Ponce *et al.* 2023).

#### Warm-up tests and evaluation of cardiorespiratory fitness: Course Navette test

For the evaluation of V̇O_2_max, the Course Navette test was used, which is a continuous incremental maximum field test performed over a 20 meter distance, with an initial running speed of 8.5 km/h and an increase of 0.5 km/h every minute. The test was stopped due to participant fatigue or when they failed to step on the 20 m line before the signal sounded twice consecutively. V̇O_2_max measurement was conducted indirectly using the Course Navette test following the method proposed by Léger *et al.* (1988), using the formula: V̇O_2_max = −24.4 + 6*X1, where X1 is the maximum speed (km/h) reached by the athlete. In addition, the meters completed by the athletes were recorded using the method proposed in García and Secchi’s work (2014) (García & Secchi 2014). All athletes were required to come early in the morning after having had their usual intake.

### Study variables

- **Hormonal variables:** progesterone (nmol/L), estrogen (pmol/L), FSH (mIU/mL), LH (mIU/mL), total testosterone (nmol/L), SHBG (nmol/L), urinary LH, progesterone/estrogen ratio (P/E) and free androgen index (FAI (total testosterone (nmol/L)/SHBG (nmol/L)) × 100.
- **Hematological and biochemical variables:** hemoglobin (g/dL), hematocrit (%), red blood cells (10^6^/μL) and iron (μg/dL), ferritin (mg/dL).
- **Variables related to cardiorespiratory fitness**: V̇O_2_max (mL/kg/min).

### Experimental overview

#### Determination of natural menstrual cycle phases

Hormonal fluctuations are most notable in three key phases of the natural menstrual cycle (de Jonge *et al.* 2019). Following the recommendations of de Jonge *et al.* (2019), this study considered the following phases:

**Phase I**: low estrogen and progesterone concentrations. This phase was considered from the first day of bleeding until day 5 (de Jonge *et al.* 2019, Elliott-Sale *et al.* 2021). The evaluation was conducted as soon as possible from the first day of bleeding, with a limit of day 5 of the cycle.

**Phase II**: detected by a positive urinary ovulation kit (LH detection). Characterized by a significant increase in estrogen and progesterone (compared to phase I). This phase was considered from a positive urinary ovulation kit until 48 h later (Elliott-Sale *et al.* 2021).

**Phase III**: estrogen and progesterone at higher levels than in previous phases. Progesterone needed to be greater than 16 nmol/L to be considered an ovulatory cycle (de Jonge *et al.* 2019). This phase was considered 7 days after detecting positive urinary ovulation (de Jonge *et al.* 2019, Elliott-Sale *et al.* 2021).

#### Verification of these phases was performed following the recommendations established in Recacha-Ponce *et al.* (2023)

- Menstrual cycle mapping: women informed the research team of the first day of their menstruation, considered day 1 of the cycle.
- Urinary LH measurement: participants performed ovulation detection tests starting on day 8 of the cycle until obtaining a positive result on the urine strip (ovulation test, Acofar, Spain). During the first visit, participants were instructed on the proper use of urinary LH detection strips. Tests were conducted at home, in the early morning, and results were sent daily to the research team via photographic evidence. Following a positive result, phase II evaluation was conducted within a maximum range of 48 h. In cases where a positive result was not obtained, tests were repeated in the next cycle. If no positive result was obtained in the second cycle, the participant was excluded from the study.
- Serum hormone analysis: levels of estrogen, progesterone, LH and FSH were determined in each phase to detect possible pathologies such as polycystic ovary syndrome (PCOS). To determine if ovulation had occurred, a minimum threshold of 16 nmol/L of serum progesterone was established (de Jonge *et al.* 2019, Elliott-Sale *et al.* 2021), determined between 7 and 9 days after detecting positive urinary ovulation. Based on progesterone levels, those with levels above 16 nmol/L were included in the natural ovulatory cycle group. A lower result included them in the group of women with natural cycles with deficient/anovulatory luteal phases (de Jonge *et al.* 2019, Elliott-Sale *et al.* 2021).

Following these guidelines, evaluations for women with natural menstrual cycles were conducted at the following intervals: first visit (phase I) (between days 1 and 5 of the cycle), second visit (phase II) (between days 12 and 16) and third visit (phase III) (between days 19 and 24 of the cycle). Assessments were conducted to measure the cardiorespiratory fitness of the athletes according to the phase of their menstrual cycle.

### Statistical analysis

Sample size was calculated based on a previous study reporting a mean V̇O_2_ of 4.49 (SD = 0.98; 95% CI (3.911, 5.069)) in phase I and 4.52 (SD = 0.90; 95% CI (3.988, 5.052)) in phase III. Considering the correlation values between V̇O_2_ (*r* = 0.83) reported by Farrell *et al.* and aiming for an 85% statistical power, a sample size of 46 subjects was estimated to detect a mean difference of 0.25 V̇O_2_ (mL/kg/min) between the follicular and luteal phases (Farrel *et al.* 1979). In one of our previous studies (Recacha-Ponce *et al.* 2023), 41 women were initially included, of which 14 had artificial cycles and 27 had natural cycles. However, in that previous study, seven participants were excluded due to the absence of ovulatory cycles. Because 33% of the women with natural cycles in the present study had anovulatory cycles, only 27 women were included for the final analysis, instead of the initially considered 41.

Statistical analyses were performed using the Statistical Package for the Social Sciences (IBM SPSS Statistics for Windows, version 29.0, IBM Corp., USA), where *P*-values <0.05 were considered statistically significant. Normal distribution of variables was verified using the Kolmogorov–Smirnov test. Since the variables did not present a normal distribution, nonparametric statistical tests were applied. Data were described using the median and interquartile range for continuous variables, and frequency and percentage for categorical variables. The Friedman test was used to analyze the evolution of parameters throughout the natural menstrual cycle (both OMC and AMC). Post-hoc comparisons were performed using Bonferroni adjustment for multiple comparisons. The Mann–Whitney U test was used to compare parameters between the two study groups. The significance of the results was additionally inferred by calculating the effect size using Cohen’s *d*, as described below (Fritz *et al.* 2011, Lenhard & Lenhard 2016): *d* ≤ 0.1 indicated a very small effect size, *d* ≤ 0.2 small, *d* ≤ 0.5 medium, *d* ≤ 0.8 large, *d* ≤ 1.2 very large and *d* ≥ 2.0 huge (Thomas *et al.* 2015).

## Results

A total of 27 female athletes participated in the research. Therefore, the women were classified into two study cohorts: women with an OMC, *n* = 20 (74%), and women with a natural cycle with a luteal phase deficiency/anovulation (AMC), *n* = 7 (26%).

Figure 1 shows a flowchart of the recruitment process and the inclusion of participants. 139 women were excluded from the study for not meeting the inclusion criteria (the most common reasons were using contraception or not fitting into levels II or III of McKay *et al.*’s framework).

### Sociodemographic description of the sample

The characteristics of the participants are shown in Table 1. No statistically significant differences were found between the two study groups.

**Table 1:** Participant characteristics. Values are presented as the median and interquartile range. The data shown refer to phase I of all groups.

Characteristics	OMC (*n* = 20)	AMC (*n* = 7)	*P*-value
Age (years)	26.55 (23–30.75)	26.86 ± 6.01 (22–34)	0.950
Weight (kg)	63.23 (54.82–69.05)	59.04 (51.10–67)	0.674
BMI (kg/m^2^)	23.06 (20.90–24.75)	21.78 (19.50–24.30)	0.507
Age at first menstruation (years)	12.15 (11–13)	12.71 (11.50–13)	0.604
Duration of cycles (days)	27.90 (26–28)	29.40 (25.50–33)	0.497
Duration of bleedings (days)	4.41 ± (4–5)	4.43 (3.50–5.50)	0.272
Years practicing sport	13.75 (7.25–19.50)	11.29 (4–17)	0.685
V̇O_2_max	41.75 (38.60–44.60)	48.44 ± 2.98 (41.60–56.60)	0.073

### Sex hormones

Table 2 shows the levels of OMC and ACM sex hormones in the different phases analyzed. Progesterone, estrogen, P/E ratio, FSH, LH, total testosterone and FAI levels differed between phases.

**Table 2:** Sex hormone levels in the natural OMC and in the natural luteal phase deficiency/anovulation menstrual. Values are presented as the median and interquartile range. Statistically significant *P*-values are shown in bold.

Parameters	Phase I	Phase II	Phase III	*P* value^§^	*P*-value/D de Cohen		
					I vs II	II vs III	I vs III
Progesterone (nmol/L)							
OMC	1.85 (1.47–2.49)^†^	6.18 (5.07–13.35)^‡^	33.91 (29.30–38.48)*	**3.56E** ^**−6**^	**<0.001/0.80**	**<0.001/1.66**	**<0.001/3.28**
AMC	1.18 (2.20–1.02)	2.26 (13.62–1.59)	2.20 (5.26–1.14)	0.156			
Estradiol (pmol/L)							
OMC	134.68 (100.84–179.94)^†^	319.41 (340.62–646.06)	499.59 (429.14–609.38)*	**8.76E** ^**−8**^	**<0.001/1.05**	0.342	**<0.001/2.05**
AMC	127.04 (208.20–41.49)	185.28 (290.10–56.42)	142.32 (176.90–56.42)	0.717			
Ratio P/E							
OMC	20 (26.84–9.59)^†^	29.10 (39.73–4.75)^‡^	71.80 (94.79–50.64)*	**5.03^−6^**	1.00	**<0.001/1.01**	**<0.001/2.16**
AMC	12.70 (25.36–5.20)	27.08 (49.75–12.22)	25.17 (46.34–11.17)	0.066			
FSH (mIU/mL)							
OMC	5.85 (5.21–6.80)^†^	4.75 (4.22–7.54)^‡^	2.65 (2.32–3.17)*	**4.33E^−6^**	1.00	**<0.001/0.83**	**<0.001/1.79**
AMC	6.20 (7.10–5.20)	5.90 (6.20–3.70)	5.50 (6.50–4.30)	0.276			
LH (mIU/mL)							
OMC	3.40 (3.21–4.92)^†^	9.10 (6.59–23.54)^‡^	3.70 (2.67–4.93)	**6.77E^−5^**	**<0.001/0.61**	**<0.001/0.62**	1.00
AMC	2.90 (14.0–1.90)	6.50 (13.90–1.50)	5.30 (13.50–2.0)	0.772			
Total testosterone (nmol/L)							
OMC	1.11 (1.03–1.38)^†^	1.33 (1.18–1.57)^‡^	1.18 (1.01–1.24)	**0.013**	0.66	**0.034/0.84**	1.00
AMC	1.08 (1.31–0.83)	1.15 (1.56–0.83)	1.11 (1.90–0.72)	0.618			
SHBG (nmol/L)							
OMC	72.30 (63.41–87.96)	82.80 (66.32–95.93)	80.05 (68.12–99.84)	0.387			
AMC	94.80 (114.20–65.80)	71.20 (97.90–53.60)	66.50 (102.20–37.0)	0.180			
FAI (nmol/L)							
OMC	1.65 (1.45–1.91)	1.63 (1.49–2.35)^‡^	1.29 (1.22–1.86)*	**0.022**	1.00	**0.022/0.46**	0.081
AMC	1.28 (3.0–0.67)	1.57 (2.91–0.92)	1.87 (5.0–0.98)	0.156			

* Significantly different from phase I.

^†^ Significantly different from phase II.

^‡^ Significantly different from phase III.

^§^ Friedman test.

OMC, ovulatory menstrual cycles; P/E ratio: progesterone/estrogen ratio; FAI, free androgen index: ((total testosterone/SHBG) × 100).

**Figure 2:**
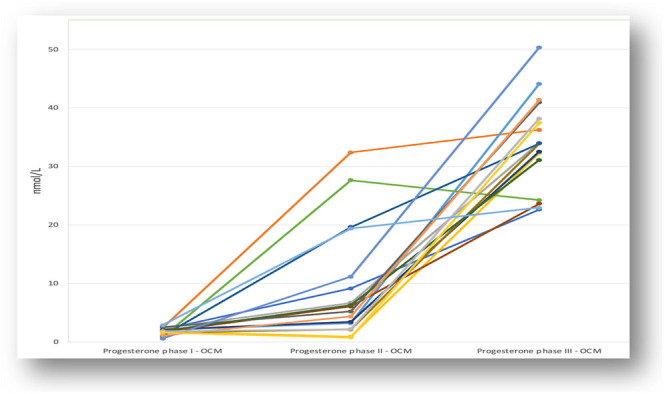
Progesterone fluctuation in OCM group: individual levels.

**Figure 3:**
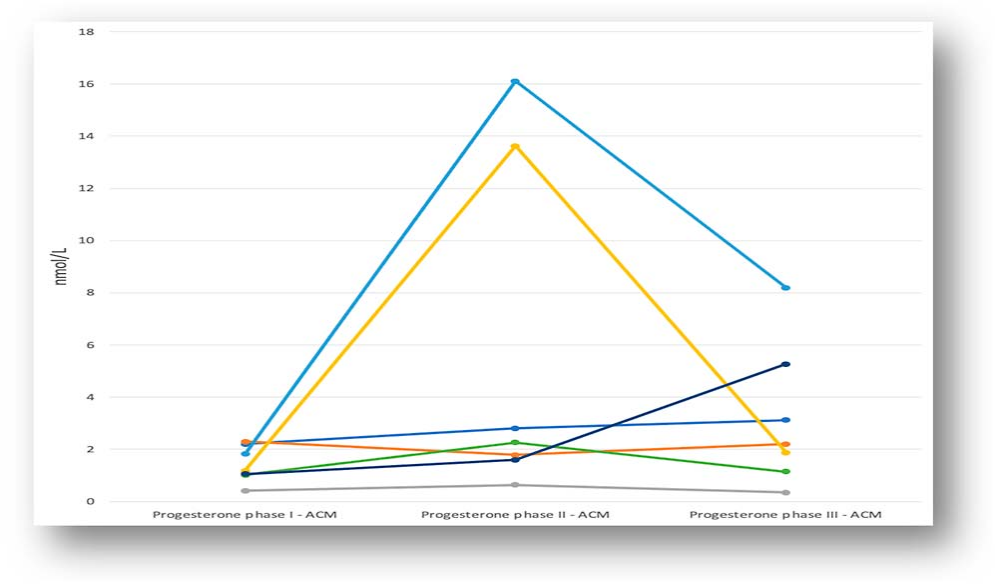
Progesterone fluctuation in ACM group: individual levels.

**Figure 4:**
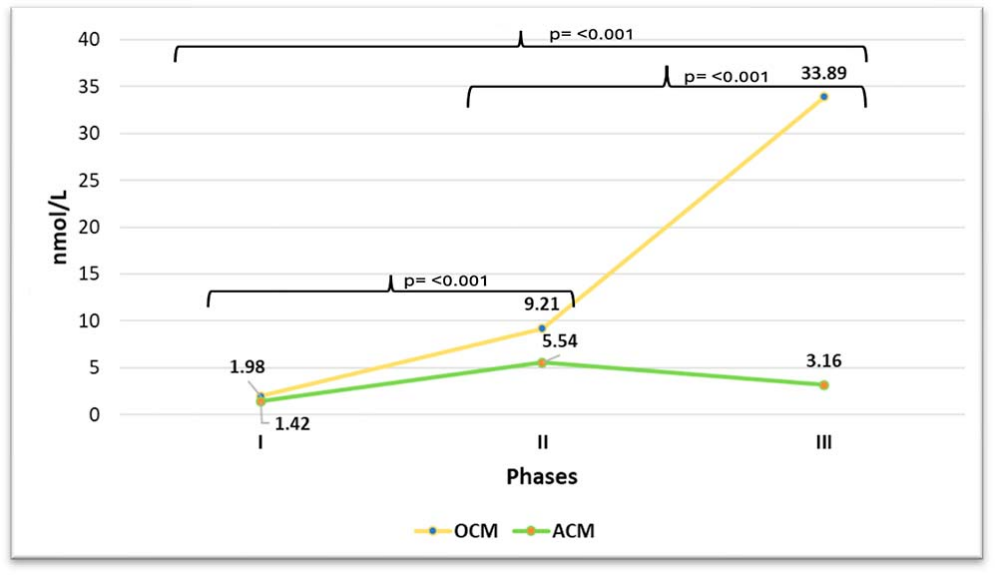
Progesterone fluctuation: OCM vs ACM.

In Figs 2, 3 and 4, the fluctuations in progesterone levels across the three phases are shown, both individually for the OCM group (Fig. 2) and individually for group 3 (Fig. 3) and grouped for both groups (Fig. 4).

### Cardiorespiratory fitness and MC phases

Table 3 shows the variables related to the level of cardiorespiratory fitness of the women with a natural MC. The V̇O_2_max shows differences between phase I compared to phase II (*P* = 0.004/*d* = 1.45) and between phase III (*P* = 0.043/*d* = 0.49). For AMC, no statistically significant differences were found.

**Table 3:** Association of the phases of natural MC and parameters of cardiorespiratory fitness. Values are presented as the median and interquartile range. Only statistically significant *P*-values are shown in bold. Only *P*-values and Cohen’s *d* values *P* < 0.05 are presented.

Parameter/group	Phase I	Phase II	Phase III	*P* value^§^	*P*-value/D de Cohen		
					I vs II	II vs III	I vs III
V̇O_2_max (mL/kg/min)							
OMC	41.60 (44.60–38.60)^†^^,^^‡^	43.10 (47.60–41.60)*	41.60 (47.60–38.60)*	**3.78E** ^**–4**^	0.004/1.45	0.429	0.043/0.49
AMC	44.50 (55.10–41.60)	44.60 (53.60–41.60)	47.60 (52.10–38.60)	0.638			

* Significantly different from phase I.

^†^ Significantly different from phase II.

^‡^ Significantly different from phase III.

^§^ Friedman test.

**Figure 5:**
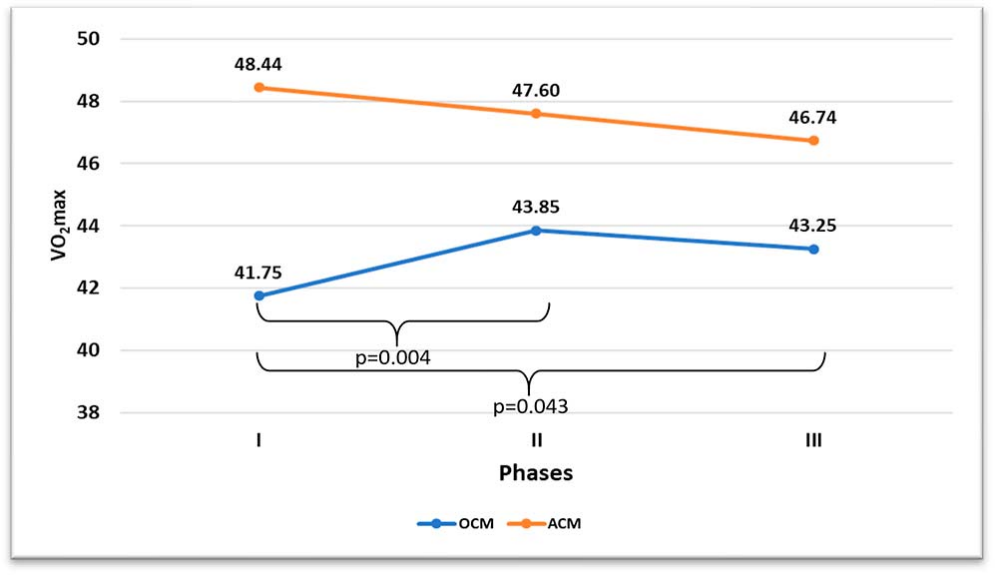
Changes in VO_2_max across key menstrual cycle phases in the OMC and AMC groups.

In Fig. 5, the evolution of V̇O_2_max in the two study groups across their key phases can be observed.

In Table 4, the results related to hematological variables of the red cell series, iron and ferritin can be observed. In the OMC group, statistically significant differences were found for the variable iron (μg/dL), which was significantly lower in phase I compared to phase II (*P* = 0.027; *d* = 0.53) and phase III (*P* = 0.005; *d* = 0.70). No significant differences were found in the remaining variables. For the AMC group, no differences were found in any variable.

**Table 4:** Hematological variables related to red blood cell series, iron and ferritin: natural MC. Values are presented as the median and interquartile range. Only statistically significant *P*-values are shown in bold. Only *P*-values and Cohen’s *d* values *P* < 0.05 are presented in bold.

Variable/group	Phase I	Phase II	Phase III	*P* value^§^	*P*-value/D de Cohen		
					I vs II	II vs III	I vs III
Red blood cells (10^6^/μL)							
OMC	4.52 (4.78–4.32)	4.53 (4.72–4.33)	4.50 (4.69–4.29)	0.450			
AMC	4.42 (4.80–4.27)	4.45 (4.71–4.43)	4.45 (4.52–4.33)	0.236			
Hemoglobin (g/dL)							
OMC	13.95 (14.57–13.00)	13.65 (14.37–13.05)	13.95 (14.20–12.85)	0.692			
AMC	13.70 (14.40–12.90)	13.60 (14.60–13.20)	13.50 (14.10–13.40)	0.236			
Hematocrit (%)							
OMC	42.20 (44.50–39.97)	41.60 (43.87–39.72)	41.65 (43.07–39.05)	0.513			
AMC	41.00 (43.60–38.80)	41.40 (43.90–40.60)	40.90 (42.10–40.60)	0.396			
Iron (μg/dL)							
OMC	54.50 (69.0–42.0)^†^^,^^‡^	82.50 (92.25–51.25)*	98.00 (112.75–64.25)*	**0.035**	**0.027/0.53**	1.00	**0.005/0.70**
AMC	102 (136–35.0)	84 (116–45)	83 (93–40)	0.368			
Ferritin (mg/dL)							
OMC	27.30 (51.0–17.20)	26.50 (42.92–13.77)	27.50 (41.62–16.40)	0.247			
AMC	14 (27.50–10)	17 (24.20–15.20)	19.10 (28.10–8.4)	0.651			

* Significantly different from phase I.

^†^ Significantly different from phase II.

^‡^ Significantly different from phase III.

^§^ Friedman test.

## Discussion

In the current literature, contradictory results are found regarding the impact of the menstrual cycle on athletic performance in women. A potential explanation for this controversy could be the lack of distinction between different types of menstrual cycles (ovulatory and anovulatory) in statistical analyses. This aspect is crucial, as evidenced in our study, where the appropriate classification of women based on their cycle type can significantly influence the results obtained.

### Distribution of cycles with deficient luteal phases/anovulation in female athletes

At the time of urinary LH detection, all women tested positive. It is important to differentiate between anovulatory cycles and cycles with a deficient luteal phase. In anovulatory cycles, progesterone levels during the luteal phase do not exceed 9.54 nmol/L (Liu *et al.* 2013). In cycles with a deficient luteal phase, progesterone levels during this phase do not reach 16 nmol/L (de Jonge *et al.* 2019). From a practical standpoint, women experience similar changes, including associated menstrual bleeding. Progesterone was the hormone used by the research team to determine whether the menstrual cycle was ovulatory, as estrogen and androgen production can vary considerably in women with ovulatory disturbances and their levels cannot be considered reference points in these disorders (Prior *et al.* 1991).

Thus, a high prevalence of female athletes with natural cycles exhibiting deficient luteal phases/anovulation is reflected (26% for the sample in this study). However, caution must be exercised regarding the results of this research at this point due to the limited sample size, indicating that many women may not be aware of their reproductive status, as their cycles appear normal on the surface. One of the reasons for this high prevalence in female athletes could be energy availability due to the high training load. This low availability leads to alterations in the menstrual cycle, potentially resulting in relative energy deficiency in sport (RED-S). This syndrome is characterized by the deterioration of physiological functions, such as reproductive function, and is primarily caused by a caloric expenditure greater than intake (Nattiv *et al.* 2007, De Souza *et al.* 2014, Mountjoy *et al.* 2014). Therefore, this group of women should be monitored independently and thoroughly, as it appears that their reproductive patterns differ from those of the general female population.

Furthermore, as mentioned throughout the paper, there are known contradictory results in the literature regarding whether the menstrual cycle influences athletic performance. A significant reason for this controversy could be the inclusion of these types of cycles in the statistical analysis without distinguishing between them (AMC vs OMC). Therefore, the presence of menstruation is not sufficient to determine the physiology or pathology of the cycle (Gimunová *et al.* 2022, Passoni *et al.* 2024); it is necessary to assess ovulation and hormonal levels, as recommended by the current literature on the correct methodology for phase verification (de Jonge *et al.* 2019, Elliott-Sale *et al.* 2021). In this way, a consensus could be reached in the future to consider the menstrual cycle when planning training loads and competitions.

### Cardiorespiratory fitness

Women with anovulatory cycles or deficient luteal phases show no variation in V̇O_2_max, unlike women with OMCs, who exhibit marked variations in V̇O_2_max. Therefore, this finding warrants further investigation, as women with deficient luteal phases or anovulation are unaware of their cardiorespiratory pattern. They experience regular menstrual bleeding, but no significant differences in the V̇O_2_max variable across different phases.

The absolute V̇O_2_max values in anovulatory women are potentially higher in all phases compared to those in the OMC. This could be caused by a high training load, which, in the long term, may lead to greater dysfunction of the menstrual cycle, thus perpetuating the dysfunctional situation. Similarly, future studies with larger sample sizes should be conducted to confirm this finding.

Examining the hormonal variables of the anovulatory menstrual cycle (AMC), no differences are observed between its phases, unlike the OMC group, which shows significant differences in estrogen, progesterone, FSH and LH. This fact could explain the lack of V̇O_2_max fluctuations in the AMC group, while fluctuations are observed in women with ovulation due to hormonal changes and their implications (Recacha-Ponce *et al.* 2023). Similarly, both total testosterone and FAI remain constant for this study group, unlike the ovulatory group. This stable and linear behavior in these variables might also contribute to maintaining V̇O_2_max without fluctuations, as testosterone has been attributed a protective role against severe fatigue (Collado-Boira *et al.* 2021).

Thus, these hormonal variations in a natural menstrual cycle could cause fluctuations in women’s fitness levels in a transient manner, with recovery and significant improvements in phases II and III. This highlights the need to adjust training, workloads and competitions according to these hormonal fluctuations that occur in the different phases of the natural OMC.

### Hematological variables, iron and ferritin

Regarding variables related to blood cell levels and the natural ovulatory cycle, no differences were found in erythrocytes, hemoglobin, hematocrit or ferritin levels. However, significant differences were observed in iron levels between phase I (59.35 μg/dL) compared to phase II (80.55 μg/dL) (*P* = 0.027; *d* = 0.53) and phase III (90.40 μg/dL) (*P* = 0.005; *d* = 0.70). These results show that, although serum iron levels decrease drastically in phase I, this does not affect hemoglobin or erythrocyte levels, and consequently, neither hematocrit. Ferritin, which reflects stored iron levels, also remains unchanged. Only circulating iron levels show variations, likely due to the effect of exercise, as it increases iron loss above baseline levels through increased iron loss in sweat, intravascular hemolysis and gastrointestinal bleeding (Peeling *et al.* 2008). In line with these findings, a study confirms that this profile of trained women has higher losses compared to sedentary women (Fogelholm 1995).

In line with our results, various studies (Chatard *et al.* 1999, Mayer *et al.* 2019, Petkus *et al.* 2019, Mullen *et al.* 2020) conclude that there is indeed a decrease in serum iron during the menstrual bleeding phase, but not a corresponding decrease in hemoglobin levels. However, the mean iron levels obtained in phase I indicate deficient serum iron levels. Iron plays a crucial role in regulating energy metabolism and oxygen transport (McDonald & Keen 1988, Clénin *et al.* 2016, Paul *et al.* 2017). There are studies showing that athletes with iron deficiency experience improvements in their cardiorespiratory fitness after iron supplementation due to greater efficiency in oxygen transport and iron utilization in the muscles (Kardasis *et al.* 2023, Solberg & Reikvam 2023). It is important to monitor these levels in female athletes, as menstruation combined with mechanical hemolysis and sweating inherent to sports practice athletes, as menstruation combined with mechanical hemolysis and sweating inherent in sports practice (Banister & Hamilton 1985) could lead to long-term hemoglobin deficiency and, consequently, iron-deficiency anemia. Barba-Moreno *et al.* (2022), consistent with our findings, confirm a decrease in serum iron during the bleeding phase (phase I) and recommend avoiding strenuous endurance training to prevent further decreases in serum iron and potential harm to hemoglobin. However, they attribute this finding to a possible decrease in hemoglobin, a notion questioned by our research, which demonstrates that while serum iron may decrease, it does not significantly affect hemoglobin levels.

Therefore, regarding its relationship with cardiorespiratory fitness, this study found that lower serum iron levels correspond to the lowest V̇O_2_max values (phase I). Therefore, fluctuations in iron levels could be a variable influencing V̇O_2_max, but it should not be considered an isolated factor, as V̇O_2_max depends on many other variables, such as the physical and psychological symptoms associated with the menstrual cycle. This presents an area for further research (Findlay *et al.* 2020).

In the case of the anovulatory cycle, all variables related to red blood cell counts, iron and ferritin showed no significant differences between phases, in line with the study by Petkus *et al.* (2019), which confirms that amenorrheic women do not experience alterations in iron levels, whereas eumenorrheic women do show changes during the menstrual cycle (Petkus *et al.* 2019). It is possible that women with anovulatory menstrual cycles (AMCs), although they menstruate monthly, may not experience as heavy bleeding as those in ovulatory cycles, which could explain the lack of differences in iron levels during phase I. Further research is needed to investigate the characteristics of menstrual bleeding in athletic women with both ovulatory and anovulatory cycles to confirm this hypothesis.

## Limitations of the study

Although the initial power analysis indicated that 46 participants were required to detect statistically significant differences in the variables of interest, our final sample included 27 athletes. In a prior phase of the study, a third group of women using hormonal contraceptives (*n* = 14) was also included. However, given our focus on analyzing natural cycles, data from this group were excluded from this article. We acknowledge that the final sample size limits the study’s statistical power and may affect our ability to detect subtle effects, particularly in the anovulatory cycle group, for which specific power was not calculated due to the lack of literature background needed to estimate an appropriate effect size. Therefore, we interpret the results with caution, recognizing that larger samples would be necessary to confirm our observations.

Furthermore, future research should investigate blood loss volume in the AMC group, associated with a potential improvement in cardiorespiratory fitness.

## Conclusion

There is a high prevalence of anovulatory cycles or cycles with deficient luteal phases among female athletes, which often goes unnoticed as these women experience regular menstrual bleeding.

Cardiorespiratory fitness shows variation in women with ovulatory cycles, in contrast to women with anovulatory cycles, who maintain stable maximum oxygen consumption throughout all phases. Similarly, the levels of sex hormones in these women do not exhibit significant changes, which could explain the stability of this variable.

Regarding hematological variables, a decrease in iron levels is observed during the bleeding phase in women with ovulatory cycles, while this variable shows no changes in anovulatory cycles. V̇O2max fluctuates in ovulatory cycles, and one of the variables responsible for this change, among others, could be iron. However, the fluctuation of V̇O_2_max depends on other factors, such as the physical and psychological symptoms associated with the menstrual cycle, leaving the possibility of an association open. It is evident that women with ovulatory cycles experience fluctuations throughout their menstrual cycle, so it may be possible to adjust training loads and competitions by reducing or modifying the type of training during menstruation and increasing them during the rest of the cycle.

Studies that do not distinguish between ovulatory and anovulatory cycles often risk diluting or even distorting the effects of the menstrual cycle on athletic performance. This omission can lead to erroneous conclusions regarding the relationship between hormonal variables, red blood cell indices, iron, ferritin and cardiorespiratory fitness. Therefore, it is essential that future studies, especially those involving female athletes with natural menstrual cycles, clearly identify the cycle type to ensure that study groups are comparable and conclusions valid.

This approach underscores the need for further research that considers the distinction between ovulatory and anovulatory natural cycles, which is essential for a better understanding of how the menstrual cycle influences physiological variables and athletic performance in women. Only through proper differentiation of groups can we obtain more accurate and applicable results.

As future lines of research, it is recommended to continue this type of study with larger sample sizes to determine the conclusions drawn more precisely.

## Declaration of interest

The authors state that this work was carried out without commercial or financial relationships that could pose a conflict of interest.

## Funding

This work did not receive any specific grant from any funding agency in the public, commercial or not-for-profit sector.

## Author contribution statement

Conceptualization was done by PR-P. CH helped with methodology. EC-B and CH helped with formal analysis. Investigation was done by PR-P, EC-B, PS-A and CH. Writing of the original draft was done by PR-P. Writing of the review and editing was done by EC-B, MM and IG-C. Visualization was done by PS-A and IG-C. EC-B helped with project administration. All authors have read and agreed to the published version of the manuscript.

## Data availability

For ethical reasons related to the preservation of patient identity, the data presented in this study are available upon request to the corresponding author.

## Ethics approval statement

Approved by the local ethical committee of the University of Jaume I (CD/77/2020): Institutional Review Board Statement: The study was carried out in accordance with the Declaration of Helsinki.

## Patient consent

All participants provided written consent before the investigation.

## Clinical trial registration

The study was registered at clinical.trials.gov (ID: NCT05576740).
